# Accessing populations of motor units

**DOI:** 10.7554/eLife.94764

**Published:** 2024-01-04

**Authors:** Eric A Kirk, Britton A Sauerbrei

**Affiliations:** 1 Department of Neurosciences, School of Medicine, Case Western Reserve University Cleveland United States

**Keywords:** electromyography, frog, hawk moth, EMG, electrode, motor systems, motor units, Mouse, Rat, Rhesus macaque, Other

## Abstract

A new device improves the way scientists can record the activity of motor units in a wide range of animals and settings.

**Related research article** Chung B, Zia M, Thomas KA, Michaels JA, Jacob A, Pack A, Williams MJ, Nagapudi K, Teng LH, Arrambide E, Ouellette L, Oey N, Gibbs R, Anschutz P, Lu J, Wu Y, Kashefi M, Oya T, Kersten R, Mosberger AC, O’Connell S, Wang R, Marques H, Mendes AR, Lenschow C, Kondakath G, Kim JJ, Olson W, Quinn KN, Perkins P, Gatto G, Thanawalla A, Coltman S, Kim T, Smith T, Binder-Markey B, Zaback M, Thompson CK, Giszter S, Person A, Goulding M, Azim E, Thakor N, O’Connor D, Trimmer B, Lima SQ, Carey MR, Pandarinath C, Costa RM, Pruszynski JA, Bakir M, Sober SJ. 2023. Myomatrix arrays for high-definition muscle recording. *eLife* **12**:RP88551. doi: 10.7554/eLife.88551.

Active, purposeful movement is a defining feature of animal life and requires precise coordination between the nervous system and muscles. Specialized nerve cells, known as motoneurons, constitute the final output of the central nervous system, and are responsible for activating muscle. Each motoneuron can innervate multiple muscle fibers, and a single electrical impulse in the motoneuron induces a corresponding impulse in these fibers, causing them to contract. Together, a single motoneuron and the muscle fibers it innervates are referred to as a motor unit (Sherrington, 1925).

Recording the electrical activity of individual motor units during voluntary contractions became possible in the 1920s with the development of needle electrodes that could be inserted into muscles. This approach has revealed many fundamental properties of the neuromuscular system by allowing indirect yet accurate and relatively non-invasive measurement of impulse times in spinal motoneurons (Adrian and Bronk, 1929; Farina and Gandevia, 2023). But it also has many limitations, such as needle electrodes getting displaced during movement and only being able to record a small number of motor units at a time. In addition, it is often challenging to assign a given impulse to a specific unit using this method because an individual electrode may detect impulses with similar electrical profiles from multiple motor units.

However, scientists can bypass some of these limitations. For example, it is possible to reliably isolate the activity of multiple individual units during movement by using a large number of electrodes organized into an array, as each motor unit will produce a unique pattern of waveforms across the electrodes. In recent decades, new techniques have been developed to record from larger numbers of motor units, including high-density electrode arrays that can be implanted into muscle, and arrays that record relatively smaller signals at many sites across the surface of the skin (Muceli et al., 2022; Negro et al., 2016).

While each of these methods has distinct strengths and limitations, there remains a need for a widely available device that is capable of the following: providing stable recordings during natural movements, when muscles rapidly lengthen, shorten, and twist; being used in a broad range of experimental preparations, muscles, and animal groups; recording many motor units simultaneously; and working for weeks or even months after implantation. Now, in eLife, Samuel Sober and colleagues at various institutions in the United States, Canada, Portugal and Germany – including Bryce Chung (Emory University) and Muneeb Zia (Georgia Institute of Technology) as joint first authors – report how they have successfully developed versatile devices called Myomatrix arrays that meet these criteria (Chung et al., 2023).

The instruments consist of four long, flexible threads that each terminate in eight electrodes (Zia et al., 2020; Lu et al., 2022). These threads can either be inserted directly into a muscle or attached to the overlying connective tissue, enabling the electrodes to move with the muscle and maintain stable recordings. Chung et al. first tested the device in freely behaving mice, showing that it provided better recordings of the activity of individual motor units than traditional wire electrodes. The team was then able to demonstrate that Myomatrix arrays could record well-isolated motor units across a wide range of muscle morphologies, species and behaviors: this included the vocal and respiratory muscles of songbirds as they breathed, the hip muscles of bullfrogs during reflex contractions, and the abdominal muscles of hawkmoth larvae undergoing a protocol commonly used in locomotion studies.

Next, Chung et al. showed that their approach could simultaneously provide stable recordings from multiple motor units, including for large muscles and during active movement (for example, for the forelimb muscles of rhesus macaques performing a reaching task). Finally, they successfully confirmed that Myomatrix arrays could be used for long-term recordings, including over at least two months after implantation in mice. Overall, this study presents a compelling validation of a novel device for high-quality, large-scale, long-term motor unit recordings in a broad range of applications.

Investigating how the nervous system controls movement often requires large-scale recordings from the motor unit population. For example, it is still unclear to what extent the brain has the flexibility to voluntarily recruit individual motor units. Under typical experimental conditions, motor units are normally activated in a fixed order as force increases, from the smallest motoneurons to the largest (Henneman et al., 1965). Even with significant training and effort, human subjects have difficulty reversing this recruitment order voluntarily, and activating larger units while suppressing smaller ones (Bräcklein et al., 2022). However, recent work in nonhuman primates suggests that the motor cortex may be capable of independently controlling individual motor units in certain contexts (Marshall et al., 2022). Recent advances in electrode design, such as the Myomatrix arrays described by Chung et al., are expected to accelerate our understanding of flexible recruitment and other major research questions in the coming years.
